# Prognostic Function and Immunologic Landscape of a Predictive Model Based on Five Senescence-Related Genes in IPF Bronchoalveolar Lavage Fluid

**DOI:** 10.3390/biomedicines12061246

**Published:** 2024-06-03

**Authors:** Cheng Zhong, Yuqiong Lei, Jingyuan Zhang, Qi Zheng, Zeyu Liu, Yongle Xu, Shan Shan, Tao Ren

**Affiliations:** Department of Respiratory Medicine, Shanghai Sixth People’s Hospital Affiliated to Shanghai Jiao Tong University, Shanghai 200230, China; zhongcheng777@sjtu.edu.cn (C.Z.);

**Keywords:** idiopathic pulmonary fibrosis, senescence, predictive model

## Abstract

Background: Idiopathic pulmonary fibrosis (IPF) is a type of interstitial lung disease characterized by unknown causes and a poor prognosis. Recent research indicates that age-related mechanisms, such as cellular senescence, may play a role in the development of this condition. However, the relationship between cellular senescence and clinical outcomes in IPF remains uncertain. Methods: Data from the GSE70867 database were meticulously analyzed in this study. The research employed differential expression analysis, as well as univariate and multivariate Cox regression analysis, to pinpoint senescence-related genes (SRGs) linked to prognosis and construct a prognostic risk model. The model’s clinical relevance and its connection to potential biological processes were systematically assessed in training and testing datasets. Additionally, the expression location of prognosis-related SRGs was identified through immunohistochemical staining, and the correlation between SRGs and immune cell infiltration was deduced using the GSE28221 dataset. Result: The prognostic risk model was constructed based on five SRGs (cellular communication network factor 1, CYR61, stratifin, SFN, megakaryocyte-associated tyrosine kinase, MATK, C-X-C motif chemokine ligand 1, CXCL1, LIM domain, and actin binding 1, LIMA1). Both Kaplan-Meier (KM) curves (*p* = 0.005) and time-dependent receiver operating characteristic (ROC) analysis affirmed the predictive accuracy of this model in testing datasets, with respective areas under the ROC curve at 1-, 2-, and 3-years being 0.721, 0.802, and 0.739. Furthermore, qRT-RCR analysis and immunohistochemical staining verify the differential expression of SRGs in IPF samples and controls. Moreover, patients in the high-risk group contained higher infiltration levels of neutrophils, eosinophils, and M1 macrophages in BALF, which appeared to be independent indicators of poor prognosis in IPF patients. Conclusion: Our research reveals the effectiveness of the 5 SRGs model in BALF for risk stratification and prognosis prediction in IPF patients, providing new insights into the immune infiltration of IPF progression.

## 1. Introduction

Idiopathic pulmonary fibrosis (IPF) is an aging-related interstitial lung disease with an extremely poor prognosis [1,2]. Patients with IPF have an overall median survival time of 3–5 years, which is almost independent of medical therapies [3]. Nevertheless, the prognosis of IPF patients varies, with some patients remaining relatively stable or progressing slowly over a prolonged period of time, while others experience a rapid decline. Previous studies have shown that patients’ gender, age, and two lung physiology variables over 12 months form a clinical model (GAP stage model) to predict the outcome of IPF patients [4,5,6]. Although this staging model has implications for prognosis prediction, it does not solve the problem of predicting outcomes for patients with similar clinical traits. Therefore, it is crucial to identify biomarkers as predictors to monitor the disease progression of IPF.

Research on biomarkers of IPF has been active in recent years, and detection of biomarkers in plasma and bronchoalveolar lavage fluid (BALF) may provide effective prognostic information. Matrix metallopeptidase 7 (MMP7) is a protein involved in extracellular matrix remodeling. Elevated levels of MMP7 were observed in BALF and plasma of IPF patients, and plasma MMP7 levels were considered a reliable biomarker of disease progression and poor prognosis [7,8]. Epithelial cell dysfunction is an important mechanism in the pathogenesis of IPF. Dysfunction leads to increased secretion of TGF-β signaling, promoting the activation of local fibroblasts and enhancing pro-fibrotic mechanisms away from the epithelium [9,10,11]. Specific biomarkers of epithelial dysfunction include mucin 5B (MUC5B), which exhibits elevated expression in terminal bronchi and honeycomb cysts. The specific genetic variation of MUC5B promoter rs35705950 correlates with a better prognosis in IPF patients [12]. Additionally, Lungen-6 (KL6) is expressed on the outer surface of alveolar epithelial type II cells and airway epithelial cells, while surfactant protein A1 (SPA) and surfactant protein D (SPD) contribute to maintaining alveolar surface tension and structural integrity. Dysfunction of epithelial cells leads to the release of SPA, SPD, and KL6 into the serum, serving as independent prognostic indicators of IPF [13,14,15]. Research on immune-related biomarkers was also expanding. Increased serum levels of C-C motif chemokine ligand 18 (CCL18), S100 calcium binding protein A12 (S100A12), and increased BALF levels of them were associated with bad outcomes [16,17,18]. Exploration of the above biomarkers indicated that IPF prognosis is associated with extracellular matrix remodeling, alveolar epithelial cell dysfunction, and immune dysregulation biomarkers.

However, the relationship between IPF prognosis and cell senescence has not been explored before. Cell senescence is a cell biological program of aging triggered by replicative telomere attrition and stress-related factors. Senescent cells may undergo cell cycle arrest, characterized by the activation of the p53 signaling pathway and the transcriptional induction of the cell cycle inhibitors p21^WAF1/CIP^ and p16^INK4a^ [7]. Emerging evidence has demonstrated that cell senescence plays a crucial role in the development of IPF. Increasing senescent alveolar epithelial cells and fibroblasts result in the accumulated secretion of multiple inflammatory proteins, known as the senescence-associated secretory phenotype (SASP). Chronic SASP creates a pro-inflammatory microenvironment that has a pro-fibrotic effect and prevents normal alveolar regeneration, further leading to the progression of disease [19,20,21]. A number of genes have been identified as markers or regulators of senescence (senescence-related genes, SRGs) based on gene manipulation experiments [22].

BALF is a convenient test for IPF patients, providing valuable insights into prognostic information through RNA expression analysis. Previous researches has investigated the potential of ferroptosis-related RNAs and hypoxia-related RNAs in predicting IPF outcomes [23,24]. However, despite being an important mechanism of pulmonary fibrosis, there have been few studies on the relationship between cellular senescence and IPF prognosis. In this study, we systematically evaluated the biomarker potential of 5 SRGs (CYR61, SFN, MATK, CXCL1, and LIMA1) in IPF BALF, which could potentially aid in predicting overall survival (OS) and pulmonary immune infiltration.

## 2. Materials and Methods

### 2.1. Exploration of SRGs

We used CellAge, a database focused on human genes associated with cell senescence, to find the latest markers and regulators involved in cell senescence [22]. A total of 279 SRGs were identified by gene manipulation experiments in different human cell types. 

### 2.2. Patient Cohort

We used the GSE70867 dataset obtained from GEO (https://www.ncbi.nlm.nih.gov/geo/ accessed on 15 March 2023 ), which includes BALF gene expression and clinical information (20 healthy controls and 176 IPF patients). The tissue sources of sequencing samples were collected in three centers (Freiburg, Siena, and Leuven), all before the initiation of systemic antifibrotic treatment. The gene expression was normalized by the R software (Version 4.2.0) “limma” package. As the data was downloaded from a public database, there was no requirement for local ethics committee approval or informed consent. The collected clinical-pathological data of the IPF patients is shown in Table S1.

### 2.3. Identification the DEGs of SRGs

The mRNA expression profile was extracted, and the batch effect was removed via the “sva” package. The differentially expressed genes (DEGs) analysis was performed through empirical Bayes statistics (“limma” package) between controls and IPF patients. A gene was considered significantly changed if the FDR < 0.05 and the gene expression changes induced aging. The DEGs in 279 SRGs were selected and visualized by the boxplot and heatmap (“ggplot2” Version 3.4.2 and “pheatmap” Version 1.0.12 packages).

### 2.4. Identification of the Prognosis Related SRGs

We used univariate Cox regression to investigate the SRGs associated with the prognosis of 176 IPF patients. *p* < 0.05 was set as the inclusion criteria. Next, a Venn diagram was performed to identify the intersection of DEGs in SRGs and prognostic SRGs, and eleven genes were screened for the next analysis. Results, including hazard ratios and the *p*-value of SRGs, were shown by the forest map. 

### 2.5. Construction and Validation of Prognostic Risk Model in IPF Patients

The 176 patients with IPF were randomly drawn into two sets at a ratio of 7 (training set):3 (testing set). We used Lasso-penalized regression and multivariate cox regression analyses to construct the prognostic model in the training set via the “glmnet” package (Version 4.1) and “survival” package(Version 3.2) of R [25,26]. Five SRGs were retained and utilized to construct a linear regression model. The risk scores of IPF patients were calculated by the expression of SRGs, and patients were classified into either high-risk or low-risk groups based on the median risk score. The distribution of risk-related groups was explored by using the “PCA” and “t-SNE” methods. Subsequently, patients were ranked in ascending order by risk scores to visualize the association between five SRG expressions and survival status.

Based on the previous steps, we used the R package “rms” to construct a nomogram and calibration curve to observe the nomogram prediction probabilities against the observed rates. Then, we utilized Kaplan–Meier (K–M) survival curves and time-dependent receiver operational characteristic (ROC) curves to further verify the accuracy of the prognostic model in training and testing sets. Next, an independent prognostic analysis was performed to validate the stability of the prognostic risk model. Stratification survival analysis was used to examine the relationship between risk outcomes and clinical information in patients with IPF.

### 2.6. Functional Enrichment Analysis

IPF patients were divided into high- and low-risk groups based on the mean risk scores of the training and test groups, and risk-related DEGs were calculated using the R package “limma”. Next, the KEGG pathway, or Gene Ontology (GO) terms, and Gene Set Enrichment Analysis (GSEA) were performed using the “clusterPofiler” R package (Version 4.0) and GSEA software (Version 4.2.2) to examine the risk-related biological functions. Normalized Enrichment Score (NES) > 1.5 and FDR < 0.05 were considered significant biological functions or pathways. The results were visualized by dot plots and enrichment maps [27,28].

### 2.7. Real-Time Quantitative PCR (qPCR) Analysis

SYBR Green-based real-time quantitative PCR (qPCR) analysis was conducted to evaluate the expression levels of six target genes (CYR61, SFN, MATK, CXCL1, LIMA1, and GAPDH) in BALF samples from 4 IPF patients and 4 control patients. Custom-designed, gene-specific primer pairs were used for amplification. Total RNA was extracted, followed by cDNA synthesis and qPCR assays with cycling conditions including an initial denaturation step at 95 °C for 10 min, followed by 40 cycles of denaturation at 95 °C for 15 s and annealing/extension at 60 °C for 1 min. Data normalization was performed using the ΔΔCt method with GAPDH as the endogenous control. The expression levels of the target genes in patient samples were presented as fold changes relative to controls.

### 2.8. Immunochemistry Analysis

Paraffin-embedded tissues were obtained from the pathology archives of 4 IPF patients and 4 control patients at the Research Ethics Committees of the First Affiliated Hospital, Zhejiang University School of Medicine. Following informed consent, transplanted lung samples were collected from each patient during surgery for tissue evaluation. The sample collection process was approved by the ethics committee. All samples were carefully chosen from distal lung tissue specimens for immunochemical analysis (Table S2).

### 2.9. Immune Cell Infiltration Analysis 

To further explore the immune infiltration landscape of IPF BALF samples, the “Xcell” package was used to estimate the 38 stromal and immune cell levels in training and testing sets [29]. Stromal and immune cell component differences between high- and low-risk groups were uncovered using the heatmap and boxplot. An analysis of the correlation between SRGs and immune cell infiltration was made using Pearson correlation analysis. KM survival analysis was applied to further explore whether the specific immune cell infiltration in IPF BALF samples could be an indicator of the poor prognosis. Then, the GSE28221 dataset was used to further validate the correlation between specific immune cells and poor prognosis, which contains 120 IPF patients’ clinical characteristics and expression profiles of peripheral blood mononuclear cells (PBMC).

### 2.10. Statistical Analysis

Statistical analysis and graph generation were performed using R statistical software version 4.1.2. For comparisons of IPF patients and healthy controls, Empirical Bayes Statistics (eBayes; Limma package) were used to estimate the expression levels of SRGs in BALF samples. The prognostic model was constructed and validated using the R packages “glmnet”, “survival”, and “survminer”. Boxplot plots and liner correlation graphs were generated using the “ggplot2” package. For the stratification analysis of clinical characteristics, we employed the Wilcoxon test to examine the relationships between the risk score and clinical characteristics. For other analyses, we used Student’s *t*-test. In both cases, a *p*-value < 0.05 was considered statistically significant.

**Figure 1:**
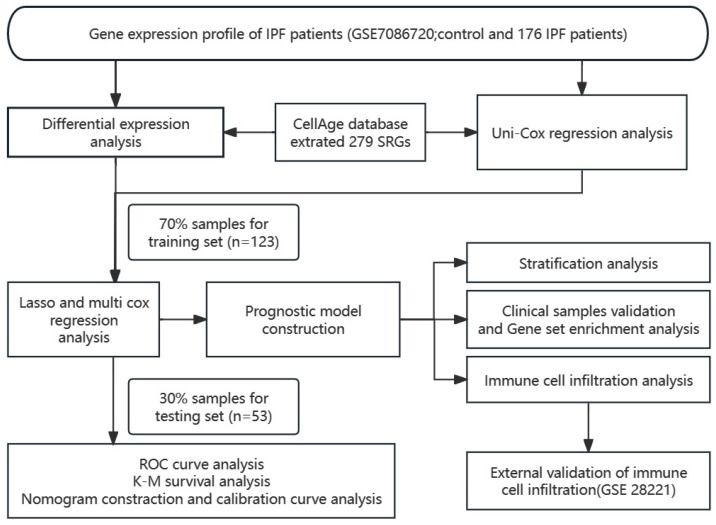
Flow chart of the present study.

The study’s flow chart was drawn to illustrate the general idea and statistical methodologies in Figure 1.

## 3. Results 

### 3.1. Identification of Prognostic SRGs in IPF

**Figure 2:**
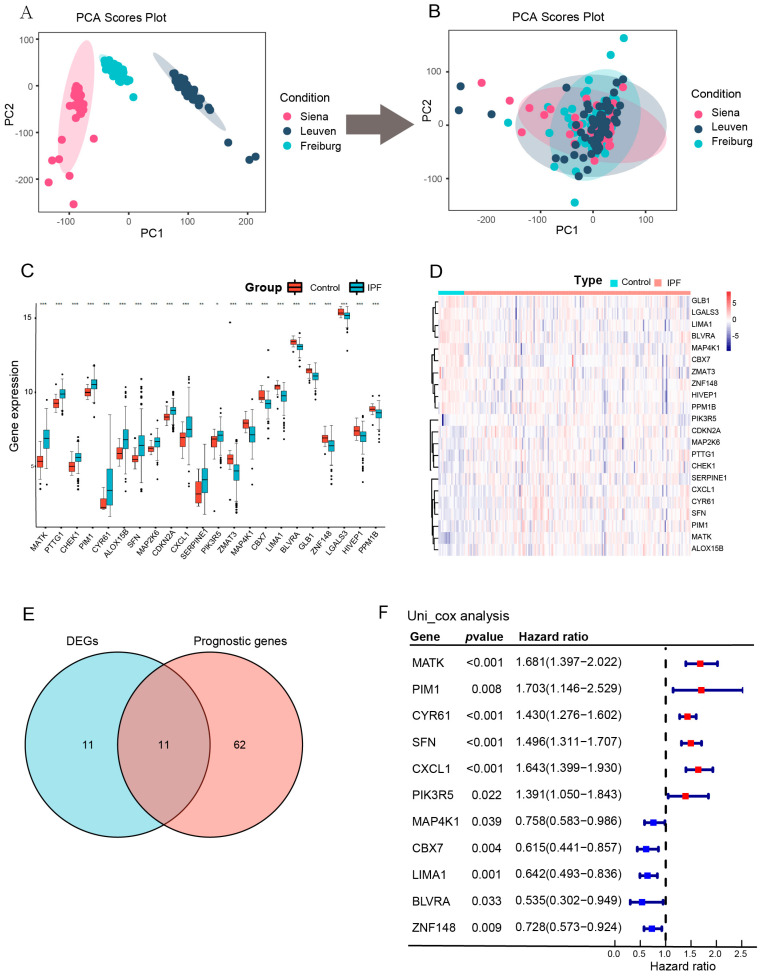
Preliminary screening for prognosis-relevant SRGs. (**A**,**B**) Eliminating the batch effect from different tissue sources. (**A**) The PCA plot before eliminating the batch effect (**B**) the PCA plot after elimination. The distance between sample points indicated how similar their RNA expression profiles were. (**C**) Comparison of 22 senescence-related DEGs in healthy volunteers and IPF patients. (**D**) The expression of the 22 senescence-related DEGs in IPF (FDR < 0.05 and the gene expression changes induce senescence). (**E**) Venn plot showing 11 overlapping genes in senescence-related DEGs and senescence-related prognostic genes. (**F**) Forrest plot of the univariate Cox regression analysis of 11 selected SRGs. Red indicates a hazard ratio > 1, representing a risk factor, while blue signifies a hazard ratio < 1, indicating a protective factor. PCA, principal component analysis; DEGs, differently expressed genes; IPF, idiopathic pulmonary fibrosis; FDR, false discovery rate; SRGs, senescence-related genes. *, **, and ***, respectively, represent the *p* values of the Wilcox test <0.05, <0.01, <0.001.

The GSE70867 dataset includes the BALF mRNA expression profile and clinical information of 176 IPF patients and 20 healthy volunteers [30]. The detailed clinical information is shown in Table S1. To comprehensively assess the mRNA expression profile of senescence-related genes (SRGs) in the GSE70867 dataset, batch effects of three tissue sources (Freiburg, Siena, and Leuven) were eliminated by using the “sva” package (Version 3.42) to identify and adjust surrogate variables (Figure 2A,B). Then, a total of 279 SRGs were downloaded from the CellAge database (https://genomics.senescence.info/ accessed on 15 March 2023) to compare IPF patients with healthy controls. Among 279 SRGs, we identified 22 differentially expressed genes (DEGs) in 279 SRGs (FDR < 0.05 and the gene expression changes induce aging) (Figure 2C,D; Table S3).

Then, we performed a univariate cox regression analysis of 279 SRGs to find overall survival (OS)-related SRGs. 73 SRGs were observed with *p* < 0.05 in univariate analysis. Subsequently, the overlap of DEGs-related SRGs and OS-related SRGs was shown as a Venn diagram (Figure 2E), and the univariate cox results of the overlapped 11 genes (MATK, PIM1, CYR61, SFN, CXCL1, PIK3R5, MAP4K1, CBX7, LIMA1, BLVRA, and ZNF148) were shown in the forest plot (Figure 2F).

### 3.2. The Construction and Validation of the Prognostic SRGs Signature

**Figure 3:**
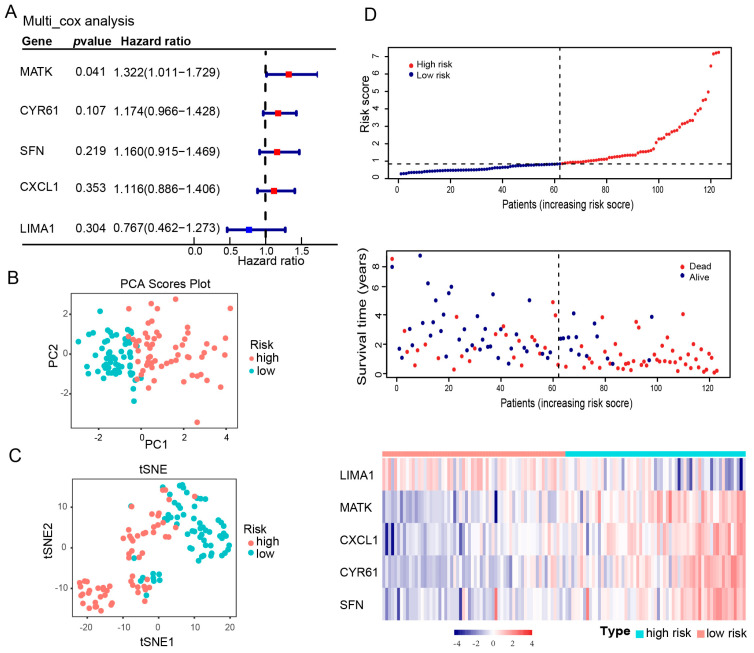
LASSO-Cox risk model construction is based on five SRGs. IPF samples were divided into high-risk and low-risk groups based on the median risk score. (**A**) Multivariate cox regression analyses of 5 prognostic SRGs that construct the risk model based on training set 123 samples. Red indicates a hazard ratio > 1, representing a risk factor, while blue signifies a hazard ratio < 1, indicating a protective factor. (**B**) PCA and (**C**) t-SNE plots show the distribution of high-risk and low-risk samples in the training group. (**D**) Risk plot, OS status of IPF patients, and heat map of expression profiles of the 5 SRGs in the training group. OS—overall survival; SRGs—senescence-related genes; PCA—principal component analysis; t-SNE—t-distributed stochastic neighbor embedding; IPF—idiopathic pulmonary fibrosis.

We randomly divided 176 IPF patients into a 7:3 ratio for training and testing sets. To reduce overfitting in the training set, we applied the LASSO-penalized Cox algorithm. LASSO, which stands for Least Absolute Shrinkage and Selection Operator, is a technique that helps in selecting a subset of the most important variables and reducing the complexity of the model. It works by adding a penalty to the size of the coefficients, effectively shrinking some of them to zero, and thus selecting only the most significant predictors. Initially, we tested 11 SRGs using the LASSO algorithm. Based on the optimal value of λ (a parameter that controls the strength of the penalty), five SRGs (MATK, CYR61, SFN, LIMA1, and CXCL1) were selected (Supplementary Figure S1). Among these, four genes were identified as risk factors, and one gene was identified as a protective factor. Next, the multivariate Cox prognostic model was constructed based on the expression of the five selected SRGs (Figure 3A). The formula for the risk score was as follows: risk score = (0.2794 × MATK) + (0.1481 × SFN) + (−0.2656 × LIMA1) + (0.1096 × CXCL1) + (0.1608 × CYR61).

Moreover, each patient’s risk score was calculated. To stratify patients into high- and low-risk groups across all datasets, we used the median risk score derived from the training set as the threshold. This approach standardizes the risk classification, ensuring consistency in the application of risk scores across both the training and testing sets. The distribution of two risk groups was shown by the PCA and t-SNE plots in (Figure 3B,C; Figure S2). Afterward, patients with IPF were ranked by risk scores to explore the relationship between risk scores and survival status. The risk plot showed that patients with higher risk scores were more likely to have a bad outcome (Figure 3D). In addition, the heat map revealed significant higher expressions of MATK, SFN, CXCL1, and CYR61 and a significant lower expression of *LIMA1* in the high-risk group compared with the low-risk group (Figure 3D and Figure S2).

**Figure 4:**
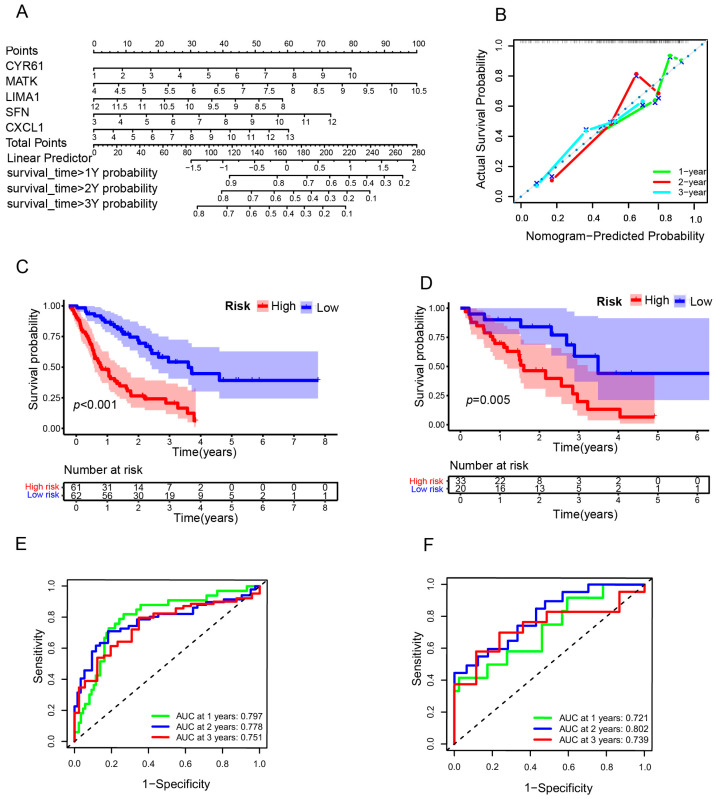
The validation of the risk model in training and testing groups. (**A**) The nomogram of the prognostic risk model based on the five SRGs. (**B**) A calibration curve was used to estimate the model fit of the nomogram. The dashed line represented the ideal nomogram, and the green, red, and blue lines represented the predicted 1-, 2-, and 3-year overall survival in IPF patients. Kaplan–Meier survival analyses of the prognostic model in training (**C**) and testing (**D**) sets. Time-dependent ROC curve analyses of risk scores in training (**E**) and testing (**F**) sets. SRGs, senescence-related genes; IPF, idiopathic pulmonary fibrosis; ROC, receiver operating characteristic.

To explore the prognostic function of the risk model, we constructed the nomogram of five SRGs for predicting the 1-, 2-, and 3-year OS of IPF (Figure 4A). The calibration curve of the nomogram revealed the goodness of fit (Figure 4B). Subsequently, the predictive power was further assessed by using the KM survival curve and time-dependent ROC curve in training (*n* = 123) and testing (*n* = 53) sets. As shown in Figure 4C,D, KM plots indicated poorer survival of IPF patients in the high-risk group than in the low-risk group (*p* < 0.001). A time-dependent ROC analysis was conducted based on the estimated risk scores of each patient. The 1-, 2-, and 3-year areas under the ROC curve (AUC) in the training set were 0.797, 0.778, and 0.751, respectively (Figure 4E). And the corresponding 1-, 2-, and 3-year AUC values of ROC curves in the testing set were 0.721, 0.802, and 0.739, respectively (Figure 4F). All the results indicated that the risk model had excellent predictive performance, with the area under the ROC curve being close to 75%.

### 3.3. Independent Prognostic Analysis 

**Figure 5:**
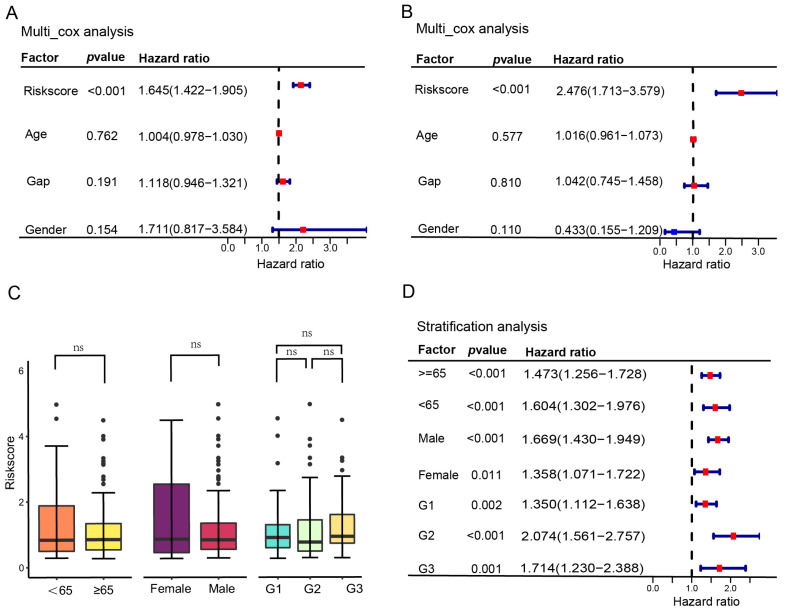
Relationships between the risk model and clinical characteristics. Multivariate cox regression analysis of clinical characteristics and risk scores in the training (**A**) and testing (**B**) cohorts. (**C**) Relationships between the risk score and clinical characteristics of IPF patients. (**D**) Stratification analyses of the predictive value of the risk score based on univariate cox regression analyses. Red indicates a hazard ratio > 1, representing a risk factor, while blue signifies a hazard ratio < 1, indicating a protective factor. ns—non-significant; IPF—idiopathic pulmonary fibrosis.

To validate the predictive stability of SRG-based risk scores, univariate and multivariate cox regression analyses of clinical characteristics and risk scores were performed in training and testing sets (Table S4). After multivariable adjustment, clinical characteristics including age, gender, and GAP stage system showed no relationship with the OS rate for IPF patients (*p* > 0.05), and only the risk scores remained an independent prognostic factor of IPF patients in training (HR = 1.645, 95% CI = 1.422–1.905, *p* < 0.001) and testing sets (HR = 2.476, 95% CI = 1.713–3.579, *p* < 0.001) (Figure 5A,B).

### 3.4. Association of Prognostic Model and Clinical Characteristics of Patients with IPF

We analyzed the relationships between risk scores and patients’ clinical parameters, including gender, age, and Gap stage. The differences in risk scores between subgroups sorted by age (≥65 years and <65 years), gender (male and female), and Gap stage (Gap Ⅰ, Gap Ⅱ, and Gap Ⅲ) were compared via the Wilcoxon test. The results showed that risk scores were not associated with age, gender, or Gap stage (Figure 5C). 

Furthermore, stratification analyses were conducted to assess the predictive ability of the prognostic model in multiple IPF subgroups via univariate cox regression and KM survival analyses. All results in subgroups were consistent with primary analyses, showing that the risk score constructed by the 5 prognostic SRGs was significantly associated with the OS rate (*p* < 0.05) (Figure 5D; Figure S3).

### 3.5. Biological Process Analysis of SRGs

**Figure 6:**
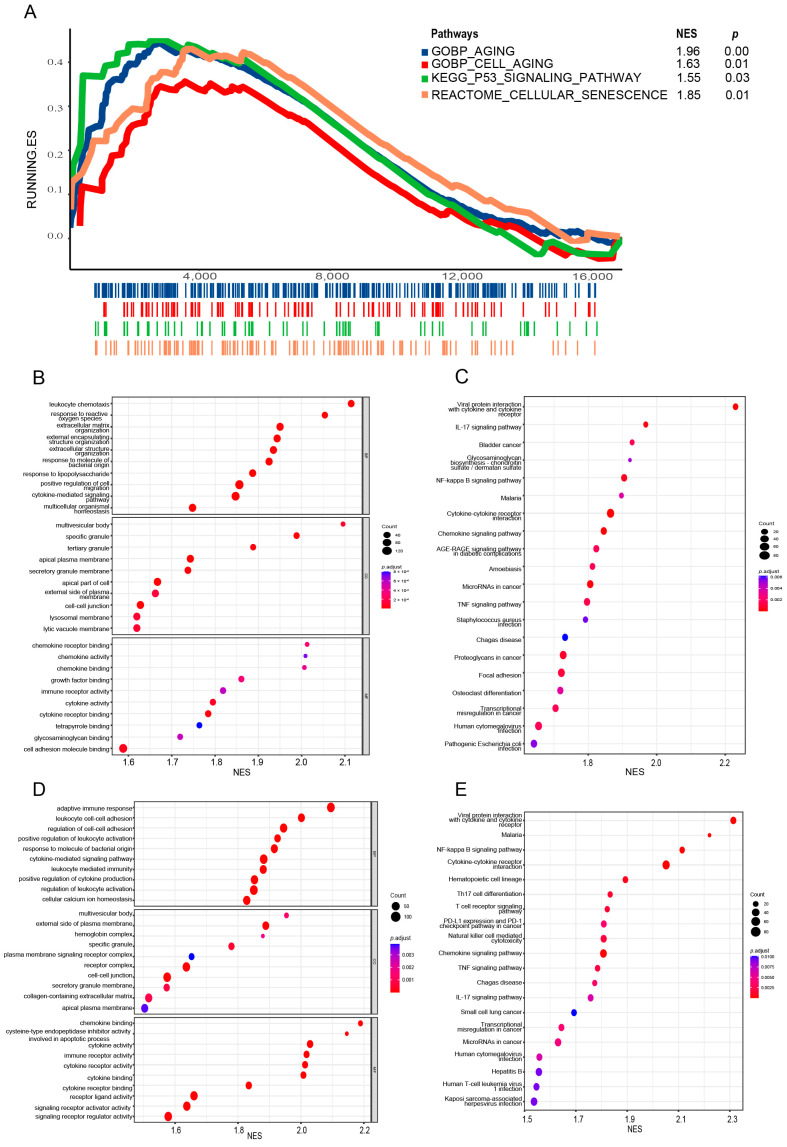
Biological analysis of 5 SRGs. (**A**) Gene set enrichment analyses of senescence-related pathways between high-risk and low-risk groups in the training set. GO (**B**,**D**) and KEGG (**C**,**E**) analyses of risk score-related genes in the training set. GO—Gene Ontology; KEGG—Kyoto Encylopedia of Genes and Genomes; NES—Normalized Enrichment Score.

Gene set enrichment analysis (GSEA) was used to compare the risk-related subgroups to investigate the potential biological processes associated with the 5 SRGs. First, cellular senescence-associated pathways, as expected, were significantly activated in IPF patients in the high-risk group (Figure 6A). Then, we found that genes up-regulated in high-risk groups (including *MMP7*, *MMP9*, *COL1A1*, *COL1A2*, *TGFB1*, *S100A12*, *CCL7*, and *CCL8*) mainly focused on pathways (using GO terms) including leukocyte chemotaxis, extracellular matrix organization, extracellular structure organization, and cytokine activity in the training and testing sets (Figure 6B,D). Furthermore, the GSEA method was also utilized in the KEGG database, and the results indicated that prognostic SRGs were related to the activation of immune, fibrosis, and cancer-related pathways such as focal adhesion, chemokine signaling pathway, the IL-17 signaling pathway, microRNAs in cancer, and transcriptional misregulation in cancer (Figure 6C,E). All the results of the GSEA analysis showed that these five SRGs expressions were positively correlated with numerous pathway activation, which may lead to the poor prognosis of IPF disease.

To further investigate the specific cell type of senescence, we found a study of senescent molecular mapping of interstitial lung disease, which explored the senescence signature of lung epithelial cells and lung fibroblasts, respectively [31]. GSEA and single-sample GSEA (ssGSEA) were performed on both gene sets (senescent epithelium and fibroblast markers) to compare enrichment scores of each risk-related subgroup. The results showed that the enrichment scores of epithelial senescence signatures in the high-risk group were significantly higher than those in the low-risk group, while there was no significant difference in the enrichment scores of fibroblast senescence characteristics. This indicated that lung epithelial cells were more likely to be the sources of five SRG changes in the IPF BALF sample (Figure S4). 

**Figure 7:**
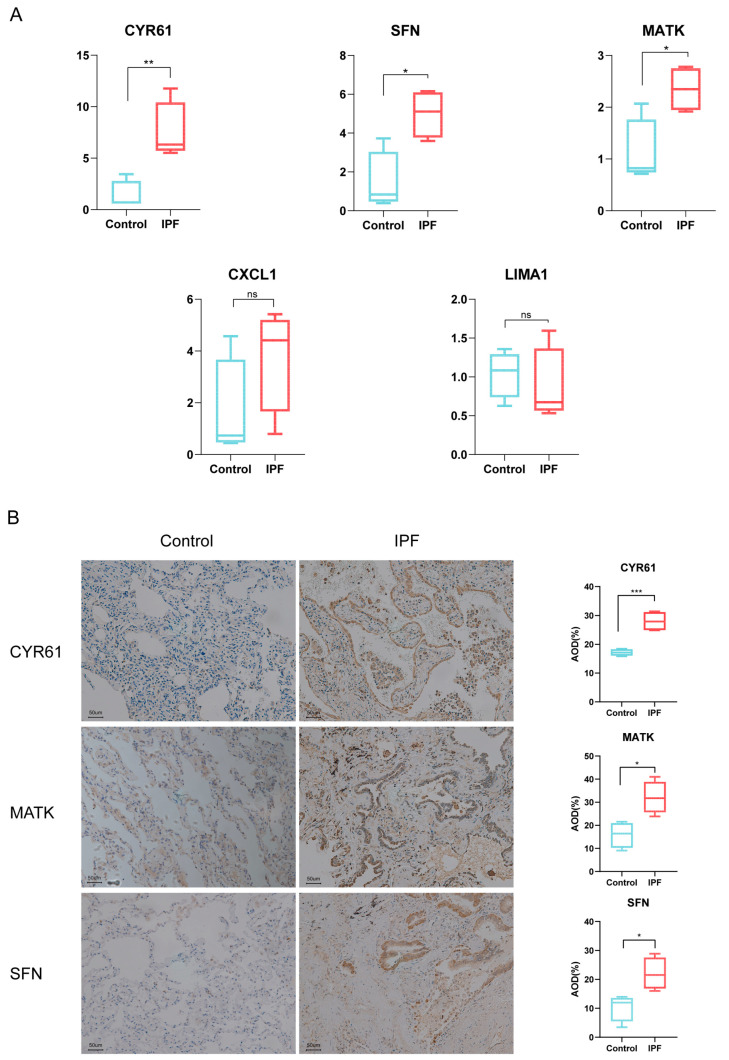
Clinical sample validation of SRGs. (**A**) qRT-PCR analysis of five SRGs in BALF samples in 4 IPF patients and 4 controls. (**B**) Immunohistochemical staining of CYR61, MATK, and SFN in 4 IPF lung tissue and 4 control lung tissue. AOD—Average Optical Density. ns—non-significan *, **, and ***, respectively, represent *p* values of *t*-tests <0.05, <0.01, <0.001.

Additionally, BALF samples were extracted from 4 IPF patients and 4 patients with pulmonary nodules. The expression differences of five SRGs between the two groups were verified through qRT-PCR sequencing. Results indicated statistically significant changes in CYR61, SFN, and MATK between the groups. Immunohistochemical staining of IPF lung tissue showed increased expression of CYR61, SFN, and MATK in distal airway epithelial cells compared to non-fibrotic lung tissue, suggesting a potential correlation between aging changes of distal epithelial cells and IPF patient prognosis (Figure 7A,B).

### 3.6. The Correlation between Immune Cell Infiltration and Five SRGs 

**Figure 8:**
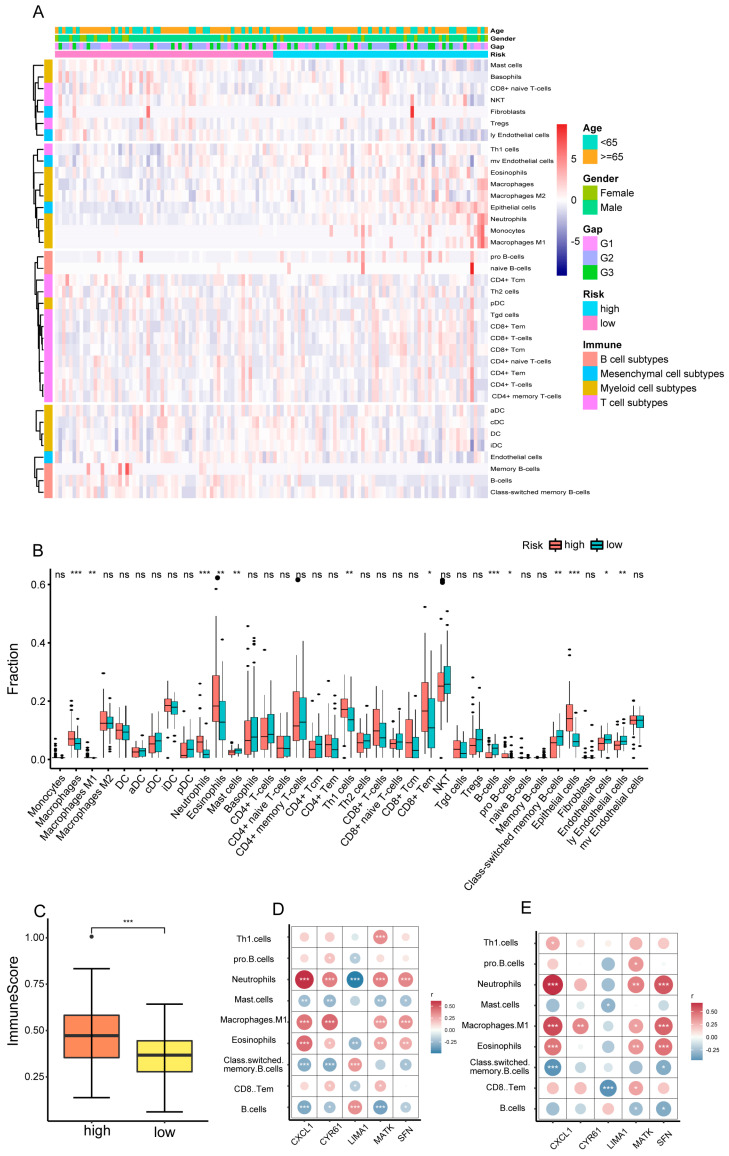
The correlation between immune cell infiltration and five SRGs. (**A**) Heatmap of immune cell subtype deconvolution using xCell based on the expression profile of IPF patients. (**B**) Comparison of compositional fractions of 37 types of cells between the high-risk and low-risk groups. (**C**) Comparison of immune scores between the high-risk and low-risk groups. (**D**) Correlation analyses of infiltration levels of nine cell types with the expression of 5 SRGs in the training set. (**E**) Correlation analyses of infiltration levels of nine cell types with the expression of five SRGs in the testing set. IPF—idiopathic pulmonary fibrosis.ns—non-significan. *, **, and ***, respectively, represent *p* values of *t*-tests <0.05, <0.01, <0.001.

Considering that the identified SRGs were closely linked to immune-related signaling pathways, we further analyzed immune cell infiltration levels in high-risk and low-risk samples in the training set. The Xcell algorithm was used to assess the immune infiltration in each BALF sample (Figure 8A–C). Patients with IPF in the high-risk group showed a remarkable increased infiltration degree of M1 macrophages (*p* < 0.01), neutrophils (*p* < 0.001), eosinophils (*p* < 0.01), Th1 cells (*p* < 0.01), CD8+ effector memory T cells (*p* < 0.05), and pro-B cells (*p* < 0.05), and a remarkable decreased infiltration degree of B cells (*p* < 0.001), class-switched memory B-cells (*p* < 0.01), and mast cells (*p* < 0.01) than those in the low-risk group (Figure 8B,C).

Next, correlation analysis was investigated between the nine immune cells described above and the prognostic SRGs selected in the training set. Results showed that the expression of *CXCL1*, *CYR61*, *MATK,* and *SFN* was positively related to the infiltration of neutrophils (*p* < 0.001), M1 macrophages (*p* < 0.001), and eosinophils (*p* < 0.05), while negatively related to mast cells (*p* < 0.05) and B cells (*p* < 0.05). For *LIMA1*, a protective factor, B cells and memory B cells were positively related to the expression level of *LIMA1* (*p* < 0.001), and the proinflammatory neutrophils were negatively related (*p* < 0.001) (Figure 8D). Then, correlation analysis was used again to validate whether the correlation between the five SRGs and the nine immune cells was stable. Although some relationships between SRGs and immune cells were not so significant, the correlation coefficient between SRGs and immune cells in the testing set showed a similar tendency as in the training set and external validation set (Figure 8E and Figure S5). In short, we demonstrated the potential role of the five selected SRGs in regulating proinflammatory activity.

Since significantly elevated levels of neutrophils, M1 macrophages, and eosinophils were found in high-risk individuals, we further explored whether the levels of these three immune cells could directly reflect patient outcomes. KM analysis showed that elevated levels of M1 macrophages and neutrophils predicted poor prognosis in both training and test sets (*p* < 0.05). Elevated levels of eosinophils also showed a worse prognosis, though this result was not significant in the training set (*p* > 0.05) (Figure S6A–F). In the validation cohort of GSE28221, elevated levels of eosinophils and neutrophils were also an indicator of a bad outcome (Figure S6G–I). The results suggested that inflammatory cell levels may be suggestive of a poor prognosis.

## 4. Discussion

Cell aging is a significant risk factor for IPF. Previous studies have demonstrated aging phenotypes in various cell types in IPF lungs, including type II alveolar epithelial cells and fibroblasts. However, limited research has investigated the correlation between cellular senescence and the prognosis of IPF. Our study conducted a comprehensive analysis of SRGs in BALF from IPF patients. We developed and validated a prognostic model based on five key SRGs and investigated their association with immune infiltration in IPF lungs. These novel findings enhance our comprehension of the relationship between senescence biomarkers and prognostic outcomes in IPF patients, underscoring their potential as predictive tools for informing clinical stratification strategies.

In this study, we constructed a prognostic risk model through five SRGs (CYR61, SFN, CXCL1, MATK, and LIMA1) and combined clinical information and immune infiltration to gain a comprehensive understanding of the predictive power of the model. First, these five selected SRGs demonstrated powerful and reliable prognostic abilities under the verification of training and testing sets. Second, we conducted a stratification analysis by clinical features to further verify the stability of the prognostic model. Moreover, as an independent prognostic factor, SRG-related risk scores exhibited good predictive function in different clinical subgroups. In addition, biological process analysis found that the strong prognostic ability of the prognostic model was probably attributed to a unique immune cell infiltration ratio, extracellular matrix remodeling and tumor-related pathway activation. We also deeply analyzed the potential contribution of senescent genes. Interestingly, high-risk patients were more likely to be enriched for epithelial cells and had a higher enrichment score in epithelial senescence signatures than low-risk patients (Figure S4; Figure 8B). In short, the above findings showed that the SRG-based classification method is a promising tool to help clinicians better understand the prognostic status of IPF patients.

The prognostic risk model was established by five SRGs, with CYR61, CXCL1, SFN, and MATK acting as risk factors and LIMA1 as a protective factor. CYR61 (cysteine-rich 61) is a member of the CCN family and is involved in the development of many diseases, including fibrosis and malignancy. The upregulated expression of CYR61 in the lung tissue induces fibroblast senescence and mediates profibrotic effects through the TGF-β1/smad3 pathway [32,33,34]. Tejaswini Kulkarni’s study found that the expression levels of CYR61 in plasma may be a predictor of the outcome of IPF patients—a higher expression of CYR61 indicates a lower OS rate [35]. Given CYR61’s role in cellular senescence and pathobiology, its predictive value and specific mechanism in the disease progression of IPF merit further study. CXCL1 (Chemokine Ligand 1 Protein) is one of the chemokines with high affinity for the CXCR2 receptor. The binding of CXCL1 to CCR2 further induces cell senescence through the p53 pathway [36,37]. Eun Kyoung Kim et al. demonstrated that normal fibroblasts can induce a senescent phenotype through autocrine CXCL1 by co-culturing with cancer cells [38]. The relationship between CXCL1 expression and lung disease prognosis has been revealed before. Aging alveolar epithelial cells (AECs) secrete high levels of CXCL1 during influenza infection, leading to excessive neutrophil recruitment. Excessive neutrophil responses induce tissue damage and exacerbate disease, resulting in a poor prognosis [39]. SFN (Stratifin, a type of 14-3-3σ protein) plays a crucial role in various cellular activities such as cell senescence, protein trafficking, DNA replication, and apoptosis [40,41]. It has been reported that SFN is induced by p53-dependent DNA damage and acts as an inhibitor of G2/M progression. SFN has also been considered a novel tumor prognostic biomarker to indicate prognosis in various cancers, including non-small cell lung cancer, pancreatic ductal adenocarcinoma, breast cancer, and ovarian cancer [42,43,44,45]. MATK (megakaryocyte-associated tyrosine kinase) is a member of the tyrosine kinase family. It phosphorylates Src kinases to make them inactive and then participates in the processes of intercellular communication, cytoskeletal organization, and cell senescence. Previous experiments have demonstrated that overexpression of MATK leads to cell growth retardation and abnormal chromosome motility [46,47]. However, the role of MATK and SFN expression in IPF tissue was not examined before. We found the upregulation of MATK and SFN expression in IPF patients’ BALF, and the degree of their upregulation was negatively correlated with prognosis. As a cytoskeleton-associated protein, LIMA1 (LIM domain and actin-binding protein 1) is important in the regulation of actin dynamics and epithelial cell junctions. RNA interference-mediated knockdown of LIMA1 is an inducer of senescence in human IMR90 cells. Additionally, the loss of LIMA1 has been associated with various cancers, as it affects cancer cell migration and invasion, thereby increasing metastatic potential [48,49,50]. Interestingly, LIMA1 expression was downregulated in BALF of IPF and was defined as a prognosis-predicting factor in our study. In conclusion, all five SRGs except LIMA1 were up-regulated in IPF BALF samples and were associated with a poor prognosis. Nonetheless, whether these genes affect the prognosis of IPF through the aging process needs further clarification.

Functional analyses found that up-regulated genes in the high-risk group were enriched in extracellular matrix organization, extracellular structure organization, chemokine activity, focal adhesion, and positive regulation of cell migration pathways, all of which are important pathways involved in the development of IPF [51,52]. Notably, we found some cancer-associated pathways activated in high-risk groups, such as proteoglycans in cancer, transcriptional misregulation in cancer, and microRNAs in cancer. These findings suggest that the aging process may be involved in both cancer pathogenesis and IPF progression [53].

Although it is unclear how immune cells play a role in IPF progression, multiple studies have shown that inflammatory immune changes in IPF can predict patients’ outcomes. Previous studies have proven that high infiltration levels of BALF neutrophils and eosinophils are directly correlated with poor OS [54,55,56], which is consistent with our results (Figure S6). Despite this relationship, it is unclear whether neutrophils and eosinophils themselves play a detrimental role or simply respond to the pathophysiological processes that lead to death. Our study showed that the increase in the number of neutrophils and eosinophils may be associated with cellular senescence, and the five SRGs were strongly correlated (Figure 8). We provided a new direction that cell senescence may link the infiltration of neutrophils and eosinophils to the increased mortality of IPF. In addition, correlations between the risk score and other immune cell infiltration were observed based on the Xcell algorithm. The results indicated that SRGs were significantly correlated with the infiltration levels of M1 macrophages, mast cells, Th1 cells, CD8+ effector memory T cells, and pro-B cells. Given the lack of studies on the relationship of these immune cells in BALF to IPF prognosis, whether cellular senescence affects the prognosis via the change of these cells requires further investigation.

In this study, we selected the transcript data of peripheral blood mononuclear cells as an external validation set to verify the correlation of immune cells with SRGs and prognosis, and the results were significant. However, the research results still have certain limitations, such as the fact that the sample size was too small and the lack of experimental evidence. Our future studies will focus on the clinical validation of this model in IPF based on our hospital resources, which may provide strong results for its timely application.

## 5. Conclusions

In conclusion, our study constructed a robust risk model based on the five SRGs by applying Lasso and multivariate cox analyses. The risk score in this model was proven to be a reliable independent prognostic index in patients with IPF. We also found that different risk subgroups had different degrees of inflammatory cell infiltration. Our results and the feasibility of prognostic prediction based on SRGs require further large sample validation for the application of clinical diagnosis and treatment.

## Data Availability

The used datasets GSE70867 and GSE28221 can be found on the GEO website (https://www.ncbi.nlm.nih.gov/geo/), and processed data can be found in supplementary tables. Other code and data can be obtained by contacting the corresponding authors if the request is reasonable.

## Supplementary Materials

The following supporting information can be downloaded at: https://www.mdpi.com/article/10.3390/biomedicines12061246/s1. Figure S1: shows the process of lasso regression to select the best lambda value. Figure S2: shows the distribution of risk scores and the expression level of five SRGs in patients in the testing set. Figure S3: uses KM curves to show that the risk-related group constructed by the SRGs has good predictive power in different clinical subgroups. Figure S4: uses GSEA analysis to show that epithelial cells are more likely than fibroblasts to be a major contributor to senescence genes. Figure S5: shows the correlation of neutrophil, M1 macrophage, and eosinophil levels with the expression of senescence-related genes (CYR61, LIMA1, and CXCL1) in the validation set GSE28221. Figure S6: shows that internal and external datasets validate the relationship between immune infiltration and prognosis. Tables S1–S4 are the clinical information of patients and data generated during processing. Each table title describes the content of the table.
