# Supplementary material for: Prognostic Function and Immunologic Landscape of a Predictive Model Based on Five Senescence-Related Genes in IPF Bronchoalveolar Lavage Fluid
Source: Biomedicines. 2024 Jun 3;12(6):1246. doi: 10.3390/biomedicines12061246 (PMC11201203; doi:10.3390/biomedicines12061246)
Supplement: Supplementary file 1 [file biomedicines-12-01246-s001.zip › Supplementary Figures.pdf]

Supplementary Figures

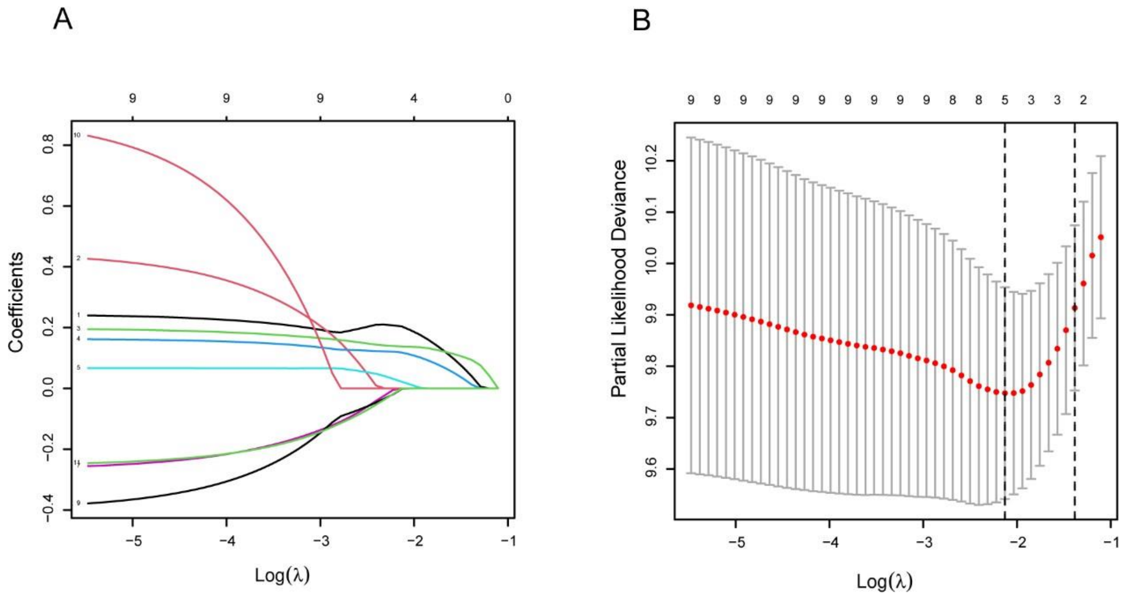

Supplementary Figure S1: (A) LASSO coefficients profiles of the 11 overlapping SRGs. (B) LASSO regression with 10-fold cross-validation obtained 5 prognostic SRGs. SRGs, senescence-related genes; LASSO, least absolute shrinkage and selection operator.

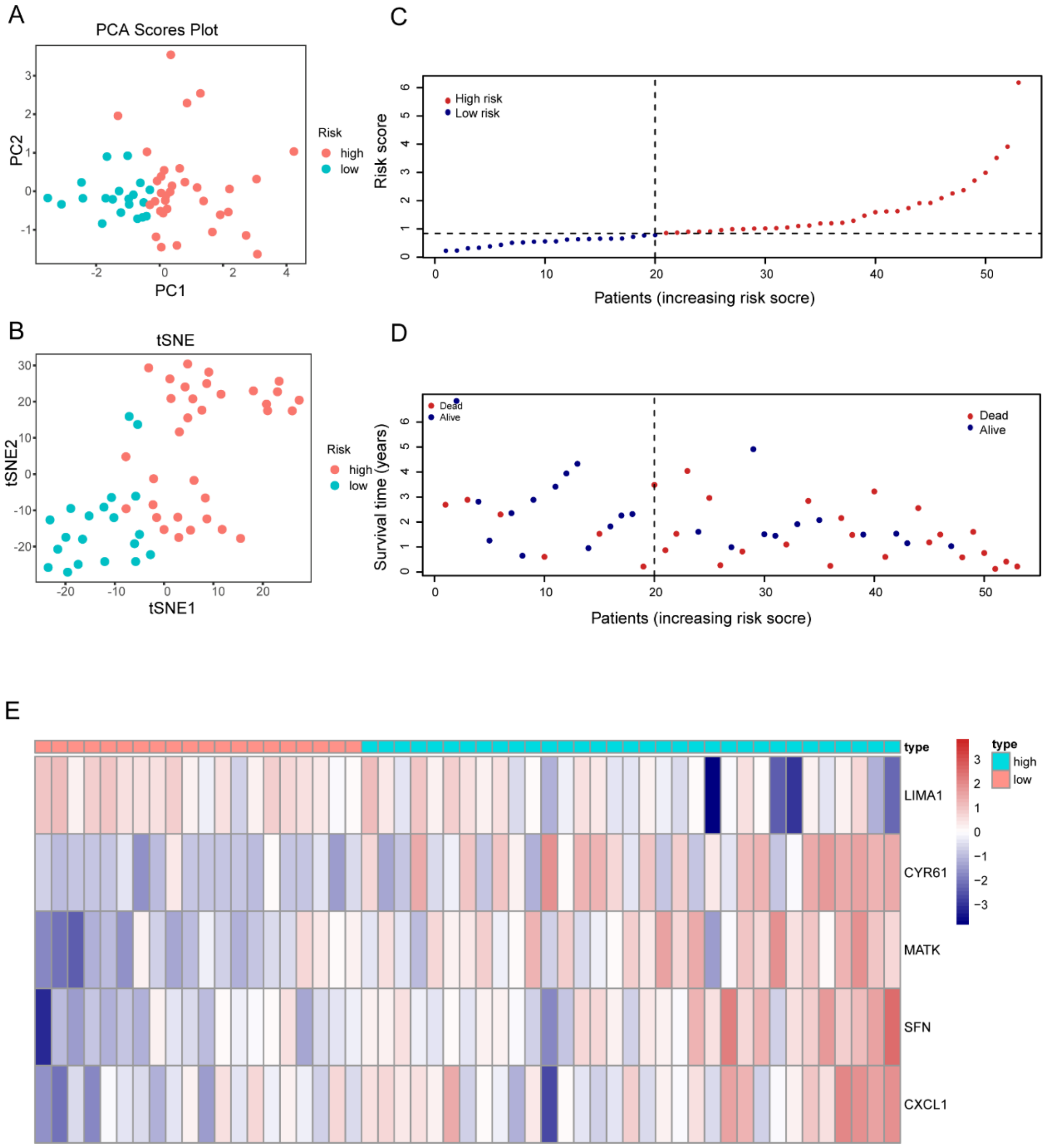

Supplementary Figure S2: IPF samples were divided into high-risk and low-risk groups in testing set. (A) PCA and (B) t-SNE plot showing the distribution of high-risk and low-risk samples in testing set. (C) Risk plot distribution, (D) OS status of IPF patients, and (E) heatmap of expression profiles of the 5 SRGs in training group. OS, overall survival; SRGs, senescence related genes; PCA, principal component analysis; t-SNE, t-distributed stochastic neighbor embedding; IPF, idiopathic pulmonary fibrosis.

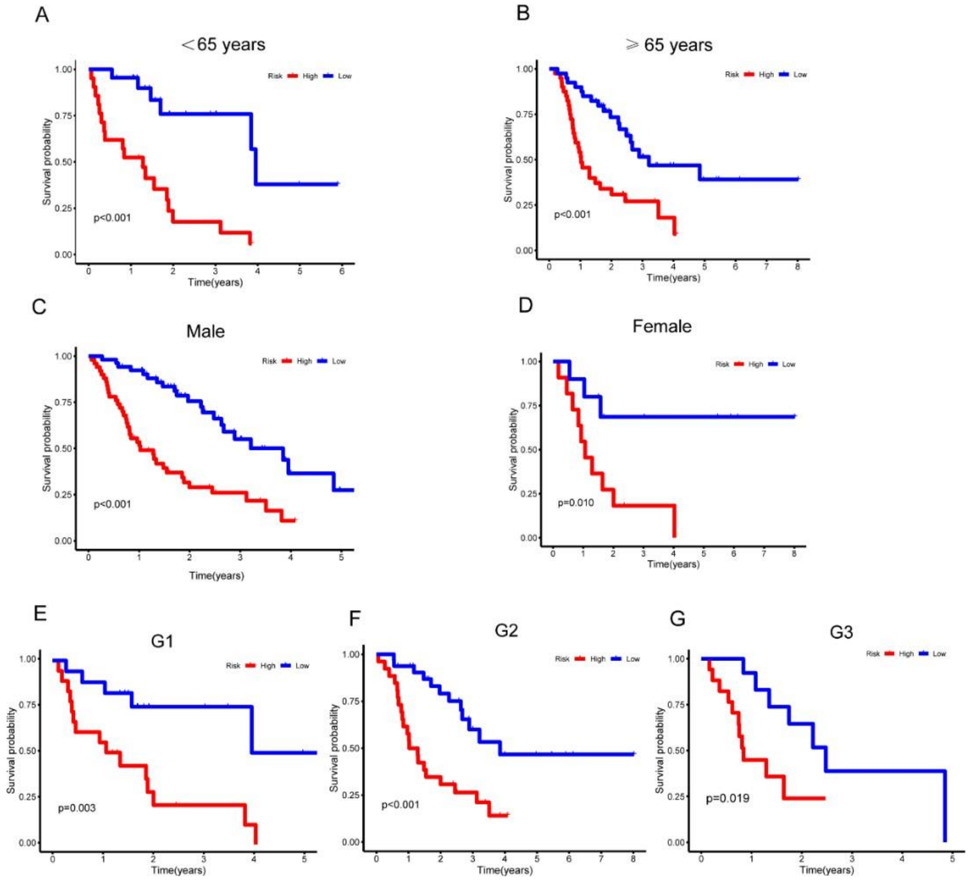

Supplementary Figure S3: The prognostic ability of the 5 SRGs for overall survival in multiple IPF subgroups. Kaplan–Meier curves for OS prediction in IPF subgroups sorted by age (＜65 years and ≥65 years), gender(male and female) and Gap stage(Gap I, Gap II and Gap III). SRGs, senescence-related genes; Gap, a staging method using gender (G), age (A), and 2 lung physiology variables (P) (FVC and Dlco).

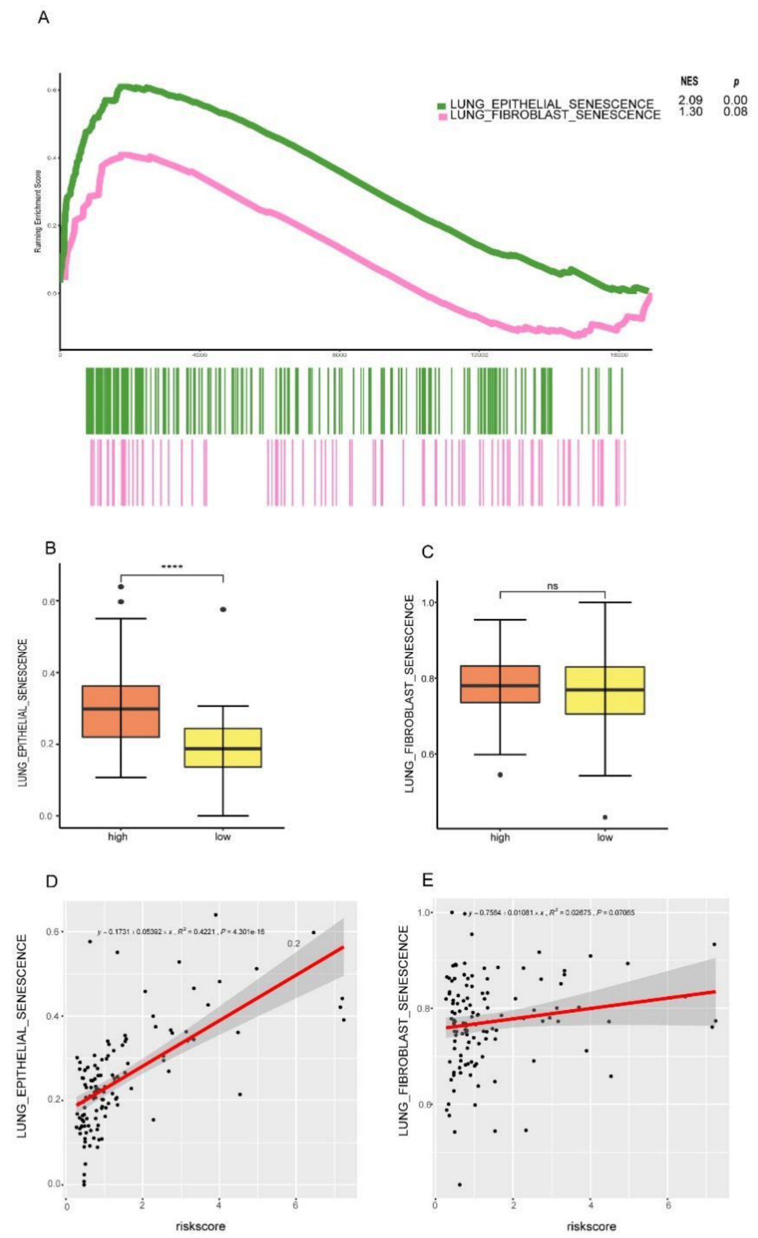

Supplementary Figure S4: (A)Comparison of the senescence enrichment scores between lung epithelial cells and lung fibroblasts based on GSEA. (B-E) Correlation analyses of the risk scores with the senescence enrichment scores of lung epithelial cells and lung fibroblasts based on ssGSEA. GSEA, gene set enrichment analysis; ssGSEA, single-sample gene set enrichment analysis. *,**, ***,**** respectively represent P values of Wilcox-test < 0.05, < 0.01, < 0.001,<0.0001. ns， none significant.

A

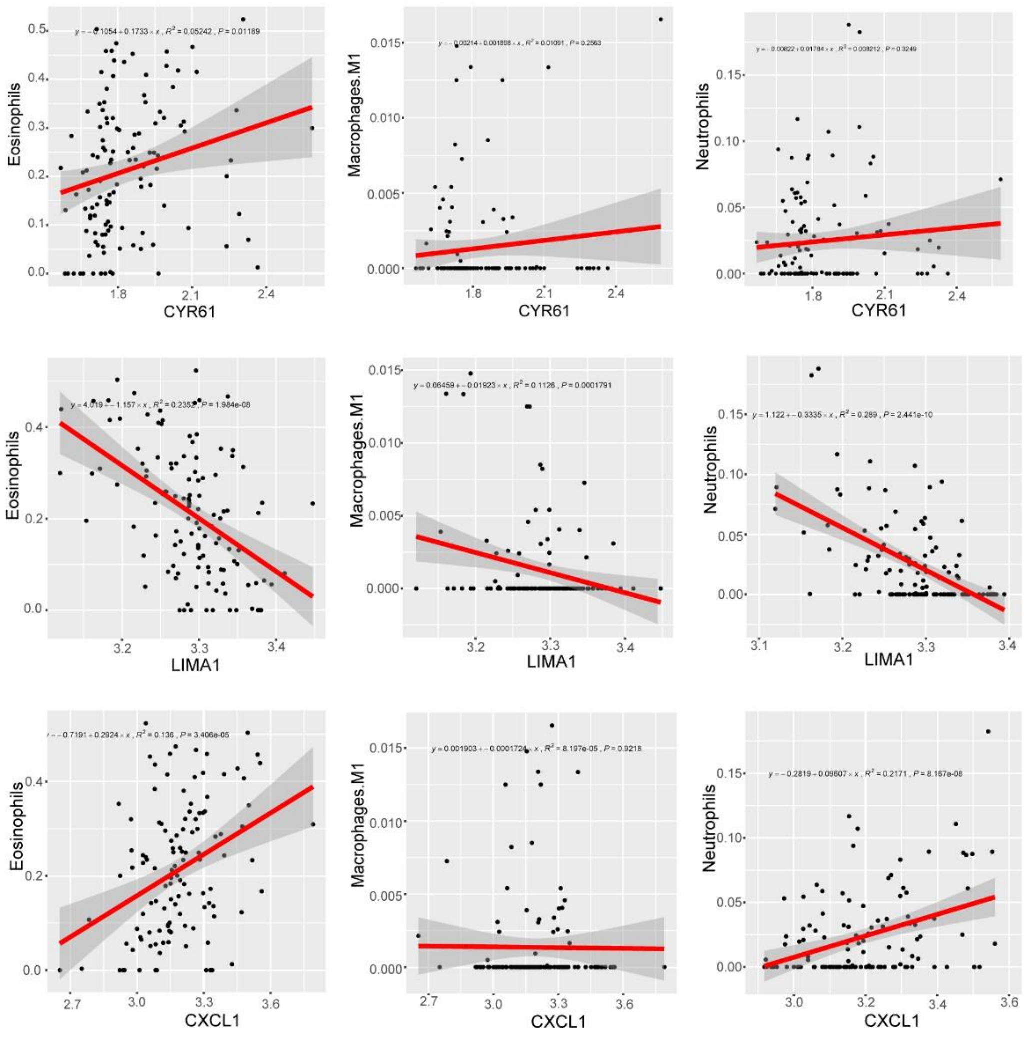

Supplementary Figure S5: Correlation analyses of the level of neutrophils, M1 macrophages, eosinophils with the expression of the senescence-related genes (*CYR61*, *LIMA1* and *CXCL1*) in validation set GSE28221.

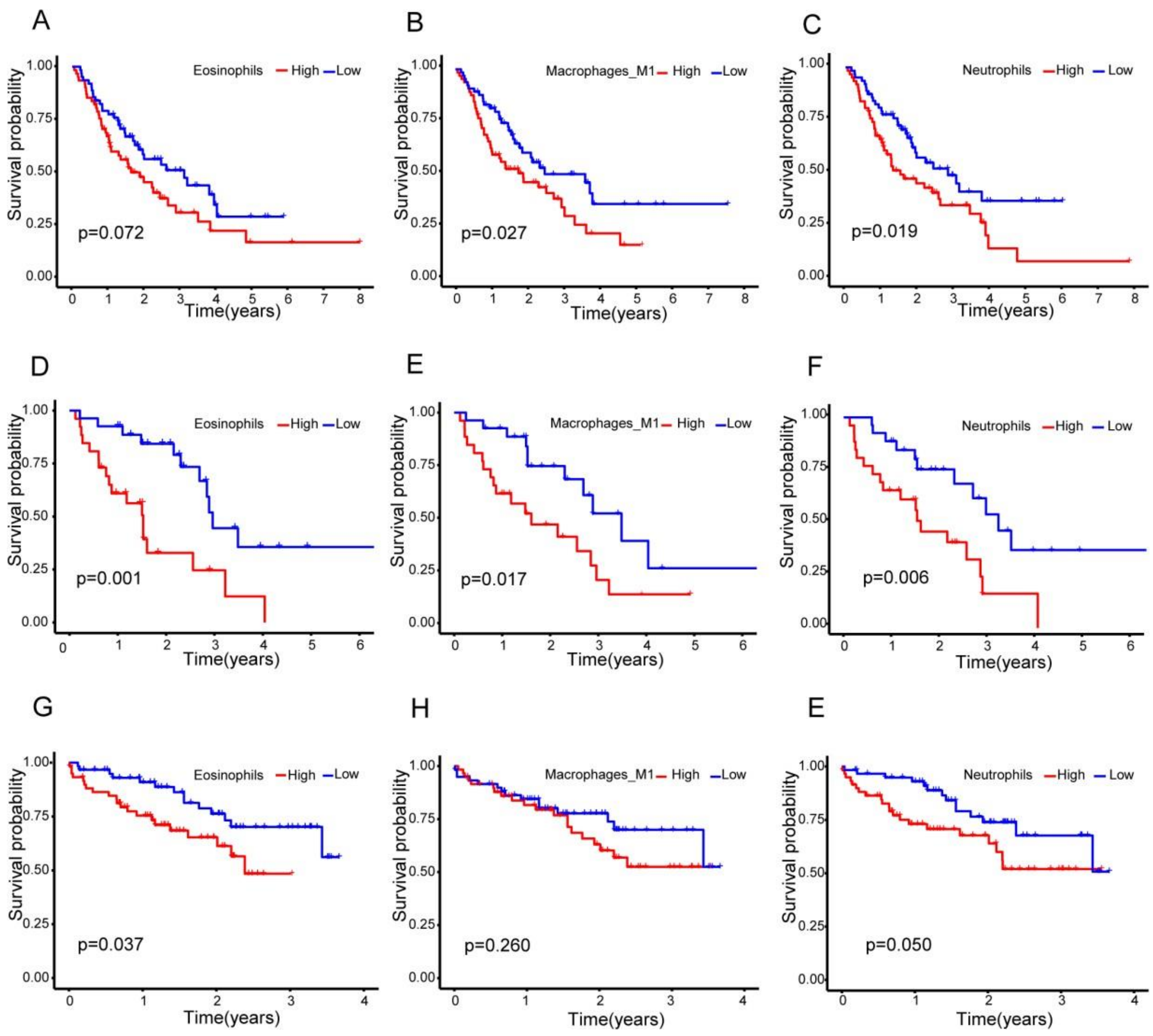

Supplementary Figure S6: Internal and external datasets validate the relationship between immune infiltration and prognosis. KM survival analyses of three specific cell types, including Eosinophils, M1 macrophages and Neutrophils in training set (A, B, C) , testing set (D, E, F) and validation set(GSE28221) (G, H, I) .KM, Kaplan–Meier.
