# Supplementary material for: Prognostic Function and Immunologic Landscape of a Predictive Model Based on Five Senescence-Related Genes in IPF Bronchoalveolar Lavage Fluid
Source: Biomedicines. 2024 Jun 3;12(6):1246. doi: 10.3390/biomedicines12061246 (PMC11201203; doi:10.3390/biomedicines12061246)
Supplement: Supplementary file 1 [file biomedicines-12-01246-s001.zip › Supplementary Table S1.pdf]

Table S1: The detailed clinical information of selected patient in GSE70867

Patient id	age	gap score	sex(0=female, 1=male)	survival_status, 0 = censored, 1 = death:ch1	survival_time,years	cohort	Group
GSM1820719	61	NA	1	NA	NA	Freiburg	Control
GSM1820720	70	NA	1	NA	NA	Freiburg	Control
GSM1820721	74	NA	1	NA	NA	Freiburg	Control
GSM1820722	55	NA	0	NA	NA	Freiburg	Control
GSM1820723	47	NA	1	NA	NA	Freiburg	Control
GSM1820724	47	NA	0	NA	NA	Freiburg	Control
GSM1820725	65	NA	1	NA	NA	Freiburg	Control
GSM1820726	50	NA	1	NA	NA	Freiburg	Control
GSM1820727	53	NA	0	NA	NA	Freiburg	Control
GSM1820728	61	NA	1	NA	NA	Freiburg	Control
GSM1820729	51	NA	1	NA	NA	Freiburg	Control
GSM1820730	61	NA	0	NA	NA	Freiburg	Control
GSM1820731	66	NA	1	NA	NA	Freiburg	Control
GSM1820732	71	NA	1	NA	NA	Freiburg	Control
GSM1820733	70	NA	1	NA	NA	Freiburg	Control
GSM1820734	62	NA	1	NA	NA	Freiburg	Control
GSM1820735	70	NA	1	NA	NA	Freiburg	Control
GSM1820736	68	NA	1	NA	NA	Freiburg	Control
GSM1820737	62	NA	1	NA	NA	Freiburg	Control
GSM1820738	61	NA	1	NA	NA	Freiburg	Control
GSM1820739	65	3	1	0	8.016438	Freiburg	IPF
GSM1820740	76	5	1	1	0.835616	Freiburg	IPF
GSM1820741	68	6	1	1	1.526027	Freiburg	IPF
GSM1820742	56	5	1	1	0.413699	Freiburg	IPF
GSM1820743	70	3	1	1	3.846575	Freiburg	IPF
GSM1820744	77	5	1	1	1.967123	Freiburg	IPF
GSM1820745	54	3	1	1	0.457534	Freiburg	IPF
GSM1820746	69	4	1	1	2.887671	Freiburg	IPF
GSM1820747	49	2	1	1	1.572603	Freiburg	IPF
GSM1820748	79	2	0	1	0.542466	Freiburg	IPF
GSM1820749	78	5	0	1	2.961644	Freiburg	IPF
GSM1820750	58	1	1	1	2.690411	Freiburg	IPF
GSM1820751	74	4	0	1	3.484932	Freiburg	IPF
GSM1820752	76	5	1	0	5.89589	Freiburg	IPF
GSM1820753	75	5	1	1	2.221918	Freiburg	IPF
GSM1820754	74	4	1	0	6.843836	Freiburg	IPF
GSM1820755	76	8	1	1	0.265753	Freiburg	IPF
GSM1820756	72	5	1	1	0.224658	Freiburg	IPF
GSM1820757	76	7	1	1	2.257534	Freiburg	IPF

GSM1820758	72	4	0	1	1.69863	Freiburg	IPF
GSM1820759	56	2	1	0	5.383562	Freiburg	IPF
GSM1820760	63	5	1	0	6.128767	Freiburg	IPF
GSM1820761	50	4	1	1	1.021918	Freiburg	IPF
GSM1820762	67	7	1	1	2.556164	Freiburg	IPF
GSM1820763	77	2	0	0	5.460274	Freiburg	IPF
GSM1820764	65	6	1	1	3.221918	Freiburg	IPF
GSM1820765	78	5	1	1	0.605479	Freiburg	IPF
GSM1820766	63	5	1	1	2.671233	Freiburg	IPF
GSM1820767	53	2	1	1	4.041096	Freiburg	IPF
GSM1820768	73	3	1	1	4.846575	Freiburg	IPF
GSM1820769	74	6	1	1	0.758904	Freiburg	IPF
GSM1820770	75	4	1	1	3.123288	Freiburg	IPF
GSM1820771	72	4	1	0	4.967123	Freiburg	IPF
GSM1820772	70	4	1	1	1.183562	Freiburg	IPF
GSM1820773	77	8	1	1	2.479452	Freiburg	IPF
GSM1820774	54	2	1	1	2.446575	Freiburg	IPF
GSM1820775	68	3	1	0	4.328767	Freiburg	IPF
GSM1820776	74	5	1	0	4.00274	Freiburg	IPF
GSM1820777	60	3	1	1	2.890411	Freiburg	IPF
GSM1820778	61	5	0	1	0.123288	Freiburg	IPF
GSM1820779	76	5	1	1	1.49863	Freiburg	IPF
GSM1820780	68	3	1	0	2.660274	Freiburg	IPF
GSM1820781	67	6	1	1	1.641096	Freiburg	IPF
GSM1820782	77	4	0	1	1.465753	Freiburg	IPF
GSM1820783	66	7	1	1	0.161644	Freiburg	IPF
GSM1820784	59	5	1	1	0.575342	Freiburg	IPF
GSM1820785	54	4	1	0	1.912329	Freiburg	IPF
GSM1820786	63	5	1	1	0.619178	Freiburg	IPF
GSM1820787	67	4	1	0	1.835616	Freiburg	IPF
GSM1820788	71	4	0	1	0.268493	Freiburg	IPF
GSM1820789	84	4	1	0	1.742466	Freiburg	IPF
GSM1820790	69	3	1	0	1.679452	Freiburg	IPF
GSM1820791	71	3	1	0	1.627397	Freiburg	IPF
GSM1820792	48	5	1	1	0.838356	Freiburg	IPF
GSM1820793	67	7	1	1	0.219178	Freiburg	IPF
GSM1820794	71	5	1	1	1.547945	Freiburg	IPF
GSM1820795	78	2	0	1	0.410959	Freiburg	IPF
GSM1820796	69	8	1	0	1.432877	Freiburg	IPF
GSM1820797	65	7	1	0	2.29863	Freiburg	IPF
GSM1820798	44	6	1	1	0.180822	Freiburg	IPF
GSM1820799	68	5	1	1	1.339726	Freiburg	IPF
GSM1820800	53	4	1	1	0.057534	Freiburg	IPF

GSM1820801	51	2	1	1	0.243836	SIENA	IPF
GSM1820802	60	1	1	0	1.509589	SIENA	IPF
GSM1820803	49	2	1	1	0.350685	SIENA	IPF
GSM1820804	83	8	1	0	1.282192	SIENA	IPF
GSM1820805	54	1	0	1	2.30137	SIENA	IPF
GSM1820806	82	7	1	1	0.539726	SIENA	IPF
GSM1820807	82	7	0	1	1.293151	SIENA	IPF
GSM1820808	68	6	0	0	2.389041	SIENA	IPF
GSM1820809	81	3	0	1	2.156164	SIENA	IPF
GSM1820810	75	4	1	0	2.887671	SIENA	IPF
GSM1820811	74	4	1	0	2.882192	SIENA	IPF
GSM1820812	62	7	1	1	1.035616	SIENA	IPF
GSM1820813	76	5	1	1	1.884932	SIENA	IPF
GSM1820814	66	6	1	1	1.350685	SIENA	IPF
GSM1820815	86	6	1	1	2.624658	SIENA	IPF
GSM1820816	70	4	1	0	2.364384	SIENA	IPF
GSM1820817	62	5	1	1	1.605479	SIENA	IPF
GSM1820818	81	3	0	0	3.027397	SIENA	IPF
GSM1820819	70	4	1	1	3.208219	SIENA	IPF
GSM1820820	78	7	1	1	0.30137	SIENA	IPF
GSM1820821	64	5	1	1	1.293151	SIENA	IPF
GSM1820822	79	6	1	1	0.380822	SIENA	IPF
GSM1820823	66	4	0	0	2.30411	SIENA	IPF
GSM1820824	62	5	1	1	0.805479	SIENA	IPF
GSM1820825	61	7	1	1	1.063014	SIENA	IPF
GSM1820826	73	8	1	1	0.871233	SIENA	IPF
GSM1820827	81	4	1	0	3.024658	SIENA	IPF
GSM1820828	68	2	0	0	3.005479	SIENA	IPF
GSM1820829	72	8	1	1	0.115068	SIENA	IPF
GSM1820830	84	4	1	1	0.756164	SIENA	IPF
GSM1820831	62	7	1	1	0.216438	SIENA	IPF
GSM1820832	72	5	1	0	2.813699	SIENA	IPF
GSM1820833	55	4	1	1	1.526027	SIENA	IPF
GSM1820834	40	2	1	0	1.808219	SIENA	IPF
GSM1820835	72	7	1	1	0.394521	SIENA	IPF
GSM1820836	73	4	1	1	0.742466	SIENA	IPF
GSM1820837	62	4	1	0	1.69589	SIENA	IPF
GSM1820838	79	3	0	0	2.353425	SIENA	IPF
GSM1820839	88	3	0	0	1.59726	SIENA	IPF
GSM1820840	62	6	0	1	2.00274	SIENA	IPF
GSM1820841	41	3	1	0	1.50411	SIENA	IPF
GSM1820842	59	5	1	0	1.334247	SIENA	IPF
GSM1820843	75	4	1	1	1.479452	SIENA	IPF

GSM1820844	79	3	1	1	0.772603	SIENA	IPF
GSM1820845	60	3	1	0	1.257534	SIENA	IPF
GSM1820846	71	3	1	1	1.167123	SIENA	IPF
GSM1820847	76	5	1	1	1.09863	SIENA	IPF
GSM1820848	71	4	1	0	1.049315	SIENA	IPF
GSM1820849	62	7	1	1	1.010959	SIENA	IPF
GSM1820850	55	4	1	1	0.668493	SIENA	IPF
GSM1820851	74	3	0	1	3.953425	LEUVEN	IPF
GSM1820852	64	2	1	0	1.608219	LEUVEN	IPF
GSM1820853	64	5	1	1	0.586301	LEUVEN	IPF
GSM1820854	58	4	1	0	0.991781	LEUVEN	IPF
GSM1820855	67	6	1	1	1.282192	LEUVEN	IPF
GSM1820856	63	4	1	1	1.852055	LEUVEN	IPF
GSM1820857	60	3	1	0	3.89589	LEUVEN	IPF
GSM1820858	64	4	1	1	4.032877	LEUVEN	IPF
GSM1820859	71	4	1	0	1.186301	LEUVEN	IPF
GSM1820860	76	3	0	0	3.482192	LEUVEN	IPF
GSM1820861	66	4	1	1	3.509589	LEUVEN	IPF
GSM1820862	65	3	1	0	1.915068	LEUVEN	IPF
GSM1820863	68	6	1	1	0.961644	LEUVEN	IPF
GSM1820864	77	4	1	0	0.89863	LEUVEN	IPF
GSM1820865	53	2	1	0	1.449315	LEUVEN	IPF
GSM1820866	64	4	1	0	2.317808	LEUVEN	IPF
GSM1820867	75	4	1	0	1.915068	LEUVEN	IPF
GSM1820868	62	2	0	0	1.147945	LEUVEN	IPF
GSM1820869	52	2	1	0	3.432877	LEUVEN	IPF
GSM1820870	72	3	1	0	1.909589	LEUVEN	IPF
GSM1820871	72	2	0	0	3.39726	LEUVEN	IPF
GSM1820872	71	7	1	1	3.819178	LEUVEN	IPF
GSM1820873	51	4	1	0	3.942466	LEUVEN	IPF
GSM1820874	60	4	1	1	1.745205	LEUVEN	IPF
GSM1820875	66	4	1	0	2.515068	LEUVEN	IPF
GSM1820876	74	6	1	0	4.978082	LEUVEN	IPF
GSM1820877	64	4	1	1	0.819178	LEUVEN	IPF
GSM1820878	69	4	1	0	1.032877	LEUVEN	IPF
GSM1820879	80	3	0	0	1.49589	LEUVEN	IPF
GSM1820880	74	4	1	0	2.076712	LEUVEN	IPF
GSM1820881	73	4	1	1	1.482192	LEUVEN	IPF
GSM1820882	75	4	1	1	0.254795	LEUVEN	IPF
GSM1820883	69	3	0	0	2.471233	LEUVEN	IPF
GSM1820884	78	5	1	1	1.082192	LEUVEN	IPF
GSM1820885	87	6	1	0	1.821918	LEUVEN	IPF
GSM1820886	61	3	0	0	3.413699	LEUVEN	IPF

GSM1820887	79	4	0	1	0.60274	LEUVEN	IPF
GSM1820888	71	4	1	1	0.586301	LEUVEN	IPF
GSM1820889	64	2	1	0	0.953425	LEUVEN	IPF
GSM1820890	52	3	1	1	0.547945	LEUVEN	IPF
GSM1820891	60	4	1	1	0.693151	LEUVEN	IPF
GSM1820892	62	2	1	0	2.260274	LEUVEN	IPF
GSM1820893	77	3	0	0	1.065753	LEUVEN	IPF
GSM1820894	73	3	1	0	1.013699	LEUVEN	IPF
GSM1820895	65	1	0	0	1.531507	LEUVEN	IPF
GSM1820896	76	4	1	0	1.572603	LEUVEN	IPF
GSM1820897	51	2	1	0	3.849315	LEUVEN	IPF
GSM1820898	78	5	1	1	0.813699	LEUVEN	IPF
GSM1820899	53	0	0	0	0.665753	LEUVEN	IPF
GSM1820900	80	5	1	0	0.652055	LEUVEN	IPF
GSM1820901	69	5	1	0	0.649315	LEUVEN	IPF
GSM1820902	77	6	1	1	0.838356	LEUVEN	IPF
GSM1820903	69	6	1	1	2.843836	LEUVEN	IPF
GSM1820904	69	2	0	0	2.452055	LEUVEN	IPF
GSM1820905	63	3	1	0	4.912329	LEUVEN	IPF
GSM1820906	65	3	1	0	1.052055	LEUVEN	IPF
GSM1820907	68	5	1	0	1.150685	LEUVEN	IPF
GSM1820908	53	2	1	0	4.084932	LEUVEN	IPF
GSM1820909	82	5	1	1	0.646575	LEUVEN	IPF
GSM1820910	79	4	1	0	3.008219	LEUVEN	IPF
GSM1820911	67	5	0	1	0.931507	LEUVEN	IPF
GSM1820912	79	7	1	1	1.994521	LEUVEN	IPF
GSM1820913	80	5	1	1	0.364384	LEUVEN	IPF
GSM1820914	68	5	1	0	2.572603	LEUVEN	IPF
