# Supplementary material for: Prognostic Function and Immunologic Landscape of a Predictive Model Based on Five Senescence-Related Genes in IPF Bronchoalveolar Lavage Fluid
Source: Biomedicines. 2024 Jun 3;12(6):1246. doi: 10.3390/biomedicines12061246 (PMC11201203; doi:10.3390/biomedicines12061246)
Supplement: Supplementary file 1 [file biomedicines-12-01246-s001.zip › Supplementary Tables S2-S4.pdf]

Table S2: Clinical samples information for immunochemical analysis

Sample type	Patient ID	Sex	Age (year)	Smoking index	Sampling location
Lung tissue	Ctrl1	Male	67	1500	the dorsal segment of the right lower lobe
	Ctrl2	Male	51	300	the posterior basal segment of the left lower lobe
	Ctrl3	Female	65	0	the anterior basal segment of the right lung
	Ctrl4	Female	72	500	the anterior basal segment of the left lung
	IPF1	Male	71	1000	the dorsal segment of the right lower lobe
	IPF2	Male	53	600	the posterior basal segment of the left lower lobe
	IPF3	Female	66	0	the anterior basal segment of the right lung
	IPF4	Female	70	0	the anterior basal segment of the left lung
BALF	Ctrl1	Male	59	1200	the left lung
	Ctrl2	Female	62	0	the left lung
	Ctrl3	Female	71	200	the right lung
	Ctrl4	Male	54	400	the right lung
	IPF1	Male	64	800	the left lung
	IPF2	Female	66	0	the right lung
	IPF3	Female	75	1000	the right lung
	IPF4	Male	54	0	the left lung

Abbreviations: Bronchoalveolar lavage Bronchoalveolar lavage fluid =BALF

Table S3: Selected SRGs (FDR<0.05 and the gene expression changes induce aging)

gene	logFC	AveExpr	t	P.Value	adj.P.Val	B	up_down	senescence_effect
MATK	1.843866	7.277752	8.003069	1.01E-13	2.54E-10	20.5863	Up	Induces
PTTG1	0.6124	10.66192	4.67611	5.40E-06	0.000324	3.740568	Up	Induces
CHEK1	0.6171	5.9525	4.4799	1.26E-	0.0006	2.9509	Up	Induces

	15	98	83	05	2	24		
PIM1	0.530242	11.41077	4.08083	6.51E-05	0.002122	1.429623	Up	Induces
CYR61	1.369028	3.855037	3.719316	0.00026	0.005868	0.155258	Up	Induces
ALOX15B	1.018788	7.293898	3.612025	0.000385	0.007681	-0.20334	Up	Induces
SFN	1.061597	6.991932	3.411497	0.000783	0.01273	-0.84889	Up	Induces
MAP2K6	0.40729	7.108012	3.126203	0.002037	0.025313	-1.71071	Up	Induces
CDKN2A	0.373117	9.45472	3.109118	0.002153	0.026193	-1.76018	Up	Induces
CXCL1	0.965114	8.230279	3.106639	0.00217	0.026317	-1.76733	Up	Induces
SERPINE1	0.801719	4.222308	3.067809	0.002458	0.02843	-1.87876	Up	Induces
PIK3R5	0.560104	7.532514	2.907071	0.004064	0.040224	-2.32644	Up	Induces
ZMAT3	-1.42193	4.996181	-4.7854	3.33E-06	0.000226	4.192248	Down	Inhibits
MAP4K1	-0.86824	7.796754	-4.49029	1.21E-05	0.000602	2.991748	Down	Inhibits
CBX7	-0.62219	10.0718	-4.39605	1.80E-05	0.000812	2.621367	Down	Inhibits
LIMA1	-0.6749	10.60248	-4.30712	2.60E-05	0.001074	2.27772	Down	Inhibits
BLVRA	-0.3399	14.28511	-4.10971	5.80E-05	0.001961	1.535728	Down	Inhibits
GLB1	-0.41168	12.08689	-4.07888	6.56E-05	0.002132	1.422477	Down	Inhibits

ZNF148	-0.63249	6.887074	-3.76969	0.000216	0.005129	0.326767	Down	Inhibits
LGALS3	-0.54576	11.57694	-3.25721	0.001324	0.018371	-1.32332	Down	Inhibits
HIVEP1	-0.51055	7.561411	-3.04067	0.00268	0.030159	-1.95588	Down	Inhibits
PPM1B	-0.35356	9.322546	-2.86116	0.004674	0.044608	-2.45027	Down	Inhibits

Table S4: The univariate and multivariate cox regression analyses of clinical characteristics and risk scores in training and testing sets

univariate cox regression analyses					
Train	HR	HR.95L	HR.95H	pvalue	
Riskscore	1.528157	1.349942	1.7299	2.05E-11	
Age	1.000821	0.976269	1.025991	0.948349	
GAP	1.054495	0.906394	1.226795	0.491973	
Gender	1.079508	0.586544	1.986786	0.805829	
Test	HR	HR.95L	HR.95H	pvalue	
Riskscore	2.435137	1.681547	3.526452	2.47E-06	
Age	1.003027	0.964235	1.043379	0.880631	
GAP	1.022363	0.821342	1.272582	0.843051	
Gender	0.601614	0.264776	1.366961	0.224948	
Multivareiate cox regression analyses					
Train	Coef	HR	HR.95L	HR.95H	pvalue
Riskscore	0.498039	1.645491	1.421604	1.904638	2.48E-11
Age	0.003973	1.003981	0.978458	1.030171	0.762333
GAP	0.111315	1.117746	0.946018	1.320649	0.1909
Gender	0.537299	1.711379	0.817216	3.583898	0.154237

Test	Coef	HR	HR.95L	HR.95H	pvalue
Riskscore	0.906726	2.476203	1.713162	3.579101	1.41E-06
Age	0.015655	1.015778	0.961405	1.073225	0.577036
GAP	0.041209	1.04207	0.744845	1.4579	0.809917
Gender	-0.83601	0.433438	0.155344	1.20937	0.110298

Coef: coefficient; HR: hazard ratio； HR.95L, HR.95H: hazard ratio 95% confidence interval.

GAP： gender, age and physiologic variables index;
